# Preparation and modeling of three‐layered PCL/PLGA/PCL fibrous scaffolds for prolonged drug release

**DOI:** 10.1038/s41598-020-68117-9

**Published:** 2020-07-07

**Authors:** Miljan Milosevic, Dusica B. Stojanovic, Vladimir Simic, Mirjana Grkovic, Milos Bjelovic, Petar S. Uskokovic, Milos Kojic

**Affiliations:** 1 Bioengineering Research and Development Center BioIRC Kragujevac, Prvoslava Stojanovica 6, Kragujevac, 34000 Serbia; 2 Belgrade Metropolitan University, Tadeusa Koscuska 63, Belgrade, 11000 Serbia; 3 Faculty of Technology and Metallurgy, University of Belgrade, Karnegijeva 4, Belgrade, 11000 Serbia; 4 Department for Minimally Invasive Upper Digestive Surgery, Clinical Center of Serbia, Hospital for Digestive Surgery - First Surgical Hospital, Dr Koste Todorovica 66, Belgrade, 11000 Serbia; 5 The Department of Nanomedicine, Houston Methodist Research Institute, 6670 Bertner Ave., R7 117, Houston, TX 77030 USA; 6 Serbian Academy of Sciences and Arts, Knez Mihailova 35, Belgrade, 11000 Serbia

**Keywords:** Biomedical engineering, Computational models

## Abstract

The authors present the preparation procedure and a computational model of a three‐layered fibrous scaffold for prolonged drug release. The scaffold, produced by emulsion/sequential electrospinning, consists of a poly(d,l-lactic-co-glycolic acid) (PLGA) fiber layer sandwiched between two poly(ε-caprolactone) (PCL) layers. Experimental results of drug release rates from the scaffold are compared with the results of the recently introduced computational finite element (FE) models for diffusive drug release from nanofibers to the three-dimensional (3D) surrounding medium. Two different FE models are used: (1) a 3D discretized continuum and fibers represented by a simple radial one-dimensional (1D) finite elements, and (2) a 3D continuum discretized by composite smeared finite elements (CSFEs) containing the fiber smeared and surrounding domains. Both models include the effects of polymer degradation and hydrophobicity (as partitioning) of the drug at the fiber/surrounding interface. The CSFE model includes a volumetric fraction of fibers and diameter distribution, and is additionally enhanced by using correction function to improve the accuracy of the model. The computational results are validated on Rhodamine B (fluorescent drug l) and other hydrophilic drugs. Agreement with experimental results proves that numerical models can serve as efficient tools for drug release to the surrounding porous medium or biological tissue. It is demonstrated that the introduced three-layered scaffold delays the drug release process and can be used for the time-controlled release of drugs in postoperative therapy.

## Introduction

A promising approach in modern medicine for drug delivery over a long time period and with a desirable rate is the use of nano-scaffolds^1–3^, and specifically electrospun-made scaffolds composed of drug loaded nanofiber mats^4–8^. Fibers are preferably prepared using a biodegradable polymer^7^, and their main function is targeted and cites specific drug delivery in human body^1–5^, without any burst release^7^, and with improved physicochemical properties^6^. This kind of drug delivery systems have provided many mechanisms that improve the therapeutic efficacy of both new and already existing drugs^7^, and may be now used for various paramedical and medical applications such as wound healing and cancer therapy^1–5^. The advantages of using biodegradable polymers for drug delivery are: no need for the second surgery to remove the scaffold once the drug is released^9^, their enhanced biocompatibility, degradability, bioactivity and resorbability^8,10–12^.

Among the most commonly used biodegradable synthetic polymers, poly(lactic-co-glycolide (PLGA) copolymer is well recognized for drug delivery processes^13^. PLGA is known for good biocompatibility and ability to achieve complete drug release^14^, which is the result of its degradation and erosion properties^15,16^. Many parameters can affect the degradation of PLGA^17^, making a release rate pattern generally unpredictable^18^. For these reasons, accurate drug release modeling can’t be achieved without incorporated PLGA degradation and erosion models^19^. It is known that both degradation and erosion change the properties of the polymer matrix: porosity and PLGA MW^14^. Those factors dramatically change the drug release nature and have to be taken into account in drug transport models^14^.

Another commonly used synthetic biodegradable polymer, poly(є-caprolactone (PCL), belongs to the group of promising biomaterials^9^ due to its biodegradability nature and biocompatibility^7^. Therefore it is used in almost all novel drug delivery systems^7,20^ , tissue engineering applications^21,22^, medical^3,8^ and paramedical^23^ devices. The advantages of using PCL in controlled drug delivery systems are its moderate drug release rate^9^ and higher degradation time^9^ in comparison to poly(lactic acid) (PLA) or PLGA biodegradable polymers. Various studies^24–26^ showed that PCL fibers are potential drug delivery options^7^, for any kind and type of drug.

The lack of the efficiency when separately using PLGA or PCL based drug delivery systems is due to an initial burst of drug release at the beginning of treatment^24^. Additionally, the main disadvantage of PCL is high hydrophobicity^7^ preventing its use in serious pharmaceutical formulations. To enhance the efficiency of drug distribution a layer-by-layer mats are proposed^27^, with an internal layer consisting the drug and outer PCL layer which thickness can control drug release process and prolong the period of the drug treatment. As shown in^28^, this technic is simple, easy to fabricate and release efficient in compare with other methods.

Many mass transport properties have to be taken into account^29^ when using polymeric material for drug release purposes^19^. Multiple experiments have shown that one of the parameter which has to be considered is the distribution of fiber radii^29–32^. The model introduced by^29^, showed that the effects of radius distribution significantly changed drug release profiles and influenced the determination of the diffusion coefficient^29^. Also, the properties of a biodegradable polymer, hydrophilic or hydrophobic drugs, and drug loading methods, can influence the final drug release profiles. In our case, to attain a good efficiency of drug delivery, it is important to choose adequate electrospinning methods for prolonged drug release, such as blending, emulsion, co-axial, phase separation, layer-by-layer, sequential electrospinning and their mutual combination^27,28,33,34^.

Computational models are very useful tool and can help in the overall development of the nanofiber drug delivery systems. The development of such computational models, which will consider the complexity of the scaffold, and accurately predict drug release rate, remains a challenge. As discussed in^35^, various approaches can be considered, from most demanding models with full 3D mesh for fibers and surrounding, to models with one dimensional (1D) radial/axial approximation of fibers, and finally to the application of smeared finite element model^36–41^. As discussed in^35^ full 3D model is impractical due to enormous number of equations to be solved over time. Alternative method with axial and radial 1D FE element is presented^36^ where axial 1D elements represent diffusion along fiber, and radial 1D element represents diffusion between fiber and surrounding continuum. Finally, in^35^ we formulated a smeared finite element which enables substitution of 1D axial and radial diffusion with continuum representation characterized by two common parameters: volumetric fraction of fibers in the system and diameters of the fibers in each node of FE mesh. Substitution is achieved by using diffusion tensor for continuum appropriately derived from 1D representation of the fibers, and both computational models are built in the FE program PAK (Program za Analizu Konstrukcija)^42^. Accuracy and applicability of those models is proved with respect to PLGA scaffold with two different ratios of PLA/PLGA constituents^35^.

The presented computational methodology is used here to simulate drug release from three‐layered PCL/PLGA/PCL fibrous scaffold. The multi-layer scaffolds consist of PLGA fibers, as an active layer that encapsulates and releases RhB molecules, and PCL fibers, as a passive layer that provides mechanical integrity and barrier properties of the scaffolds. The main goal is to achieve drug burst 21 days after surgery, which is the optimal period of postoperative treatment.

## Materials and methods

### Materials

Poly(lactide-co-glycolide) (Mw 40,000–75,000 g/mol), poly(ε-caprolactone) (Mw 80,000 g/mol), Rhodamine B (RhB), span 80, *N*,*N*-dimethylformamide (DMF), dichloromethane (DCM) and chloroform (CHCl_3_), were purchased from the Sigma-Aldrich Co. (Milwaukee, WI). The chemicals were used without further purification. Phosphate buffered saline solution (PBS) was made by dissolving one tablet of PBS, supplied by Fisher Scientific, USA, in 200 ml of deionized water (DI).

#### Preparation of a three‐layered fibrous scaffold produced via emulsion/sequential electrospinning

In this experimental part, we designed a unique three‐layered fibrous scaffold capable of a prolonged release of a hydrophilic model drug. The scaffold consists of three-layers prepared by sequential electrospinning, where the first and third layers are fabricated using poly(є-caprolactone). The second layer was produced by emulsion electrospinning using poly(lactic-co-glycolic acid) (PLGA 65:35) and Rhodamine B. For emulsion electrospinning, 3 g of PLGA were dissolved in the mixture of solvents chloroform/DMF (8.25/2.75 g), forming a 24 wt% PLGA solution. After that, Span 80 (50.0 mg) was added to this polymer solution as emulsifier. First, the Span 80/PLGA solution was magnetically stirred at 200 rpm at room temperature for 24 h. Then the 5 wt% of RhB aqueous solution (60 µl) was slowly dropped into the polymer solution to form a w/o emulsion. The mixture was additionally stirred for 2 h. The emulsion was transferred to a 20 ml plastic syringe, which had a stainless steel needle (1 mm inner diameter), and then subjected to an applied voltage 20 kV. The distance between the needle and copper collector was 10 cm and the solution feeding rate was maintained at 3 ml/h. The second polymer solution is 10 wt% poly(ε-caprolactone) in an 80:20 mixture of DCM and DMF. PCL solution was loaded into a syringe and electrospun at 20 kV and a flow rate of 2 ml/h, with a distance between the needle and the collector of 10 cm. The outer diameter of the syringe needle was 1 mm. In the sequential electrospinning process, the three-layer scaffold was designed as follows: first, the PCL fibrous layer was electrospun for 90 min, the second layer of PLGA fibers containing RhB was electrospun for 60 min and then cut at dimensions 2.5 × 3 cm. Two samples of the same dimensions are placed on the first PCL layer. At last, another PCL layer similar to the initial PCL layer covered the surface of the PCL/PLGA fibrous layers, and then two three-layer scaffolds were cut at dimensions 3 × 4 cm. The final layered sample was labeled as PCL/PLGA/PCL. The resulting three micro fibrous layers were stacked and fixed from all sides of the fibrous scaffold, which also facilitated the handling of the multi-layered scaffold. Only the edges of the scaffolds were closed with mini vacuum polymer sealer. The addition of a closed edge of the PCL layer in the multi-layer structures prevented the shrinking, improved mechanical integrity of the fibrous scaffold and act as a barrier to delay the burst release of the RhB model drug^43^.

The vertical electrospinning device (Linari Engineering, Italy) used for scaffold preparation consisted of a syringe pump (R-100 E, RAZEL Scientific Instruments), a high-voltage DC power supply generator (PCM50P120, Spellman USA). Details are given in Supplementary Fig. S1. All the electrospinning process was conducted at room temperature (23–25 °C) and a relative humidity of 45–50%, within a closed chamber.

### Drug release measurement

Rhodamine B is used as the replacement for drug, and its concentration from the fibrous scaffold is measured with a UV spectrophotometer (UV Shimadzu 1700, Shimadzu Corporation, Kyoto, Japan) at λmax = 554 nm. As a release medium for drug loading scaffold, 20 ml of PBS (phosphate buffer saline, pH 7.4) is used, and three-layered scaffold with dimensions of 3 cm × 4 cm was dipped in this solution at 37 °C. The cumulative amount of RhB released from PLGA/RhB fiber layer through PCL layers was calculated as a function of time. The experimental release of RhB from PLGA (65:35) fiber layer was investigated and is presented in^35^.

### Fiber distribution measurement

**Figure 1:**
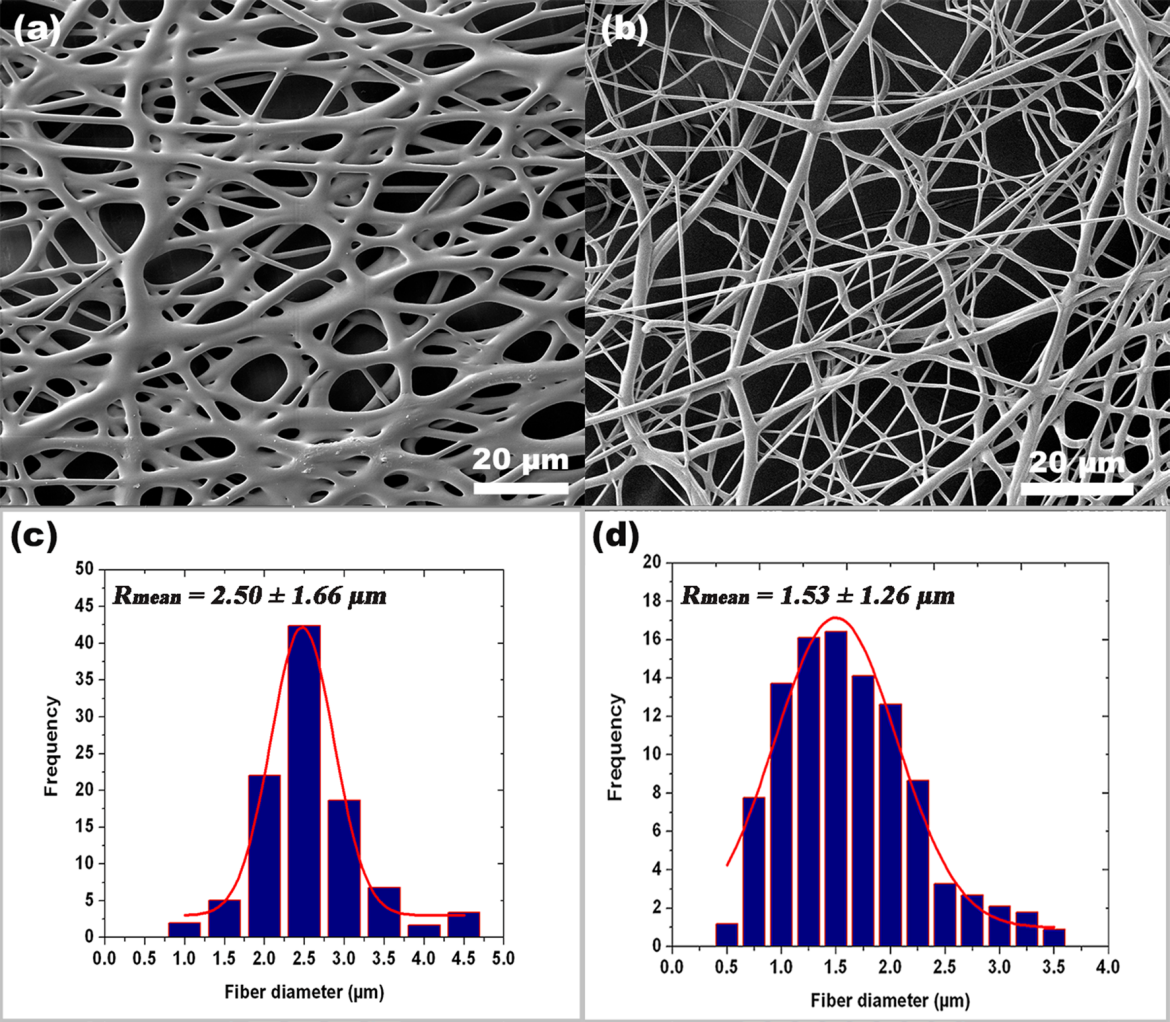
SEM image of the PLGA and PCL electrospun fibers (**a**,**b**) and the respective fiber diameter distribution histogram (**c**,**d**).

Fiber radius distribution is obtained by investigating the topology of experimentally fabricated PLGA and PCL layers. The surface structure and cross-sectional morphology were observed by field emission scanning electron microscopy (FE-SEM), Tescan Mira3 XMU (Brno, Czech Republic). The approximate shape of the fiber radius distribution is obtained by measuring the size of a statistically significant number of fibers on SEM images, as in^29^. The average diameter and fiber radius distribution of the fibers were measured from the SEM micrographs using image analysis software (Image-Pro Plus 6.0., Media Cybernetics). The diameter of the electrospun fibers was in the range of 2.50 ± 1.66 µm and 1.53 ± 1.26 µm, from PLGA and PCL fibers, with mean values of the thickness of 160 ± 1.9 μm and 200 ± 5.7 μm, respectively. Obtained fiber radius distribution is further used in our computational model. The topography of the produced polymeric fibrous scaffold was studied^35^ and the fiber diameter distribution histogram was built (as shown in Fig. 1).

As an example of fiber distribution, the approximate normal distribution was considered, as shown in Fig. 1c,d. Parameter which can be used in numerical model for stochastic distribution of radii within the model, according^31^ are: (R_0_ = 1.0 µm, R_n_ = 4.0 µm, R_mean_ = 2.5 µm) for PLGA, and (R_0_ = 0.5, µm, R_n_ = 3.5 µm, R_mean_ = 1.5 µm) for PCL.

## Computational models

### Fundamental equations

Here we summarize the basic equations from^35^ for diffusion and degradation, and formulation of the 1D and composite smeared finite element used in the computational model. The diffusion model consists of fibers and surrounding fluid, where fibers are approximated by radial 1D elements while surrounding fluid is modeled using 3D continuum elements. The balance equation for diffusion in a 3D space, which is based on Fick’s law, can be written as^44^:

1 $$ - \frac{\partial c}{{\partial t}} + \frac{\partial }{\partial x}_{i} \left( {D_{{{\text{ij}}}}^{{}} \frac{\partial c}{{\partial x_{j} }}} \right) + q = 0,{\text{ sum on }}i,j; \, i = 1,2,3 $$

where $c$ is concentration, $D_{ij}$ are diffusion tensor coefficients, and $q$ is a source term. The effects of degradation and erosion of nanofibers occurring during drug release are included in our model in accordance with^14^. The modified diffusion coefficient of drug release through a PLGA polymer fibers can be written as D = D(M_w_,φ), where M_w_ is the average molecular weight (MW) of PLGA and ϕ is porosity. Diffusion coefficient function D = D(M_w_, φ) is given as:

2 $$ D = \frac{{(1 - \phi )D_{s} + \kappa \phi D_{l} }}{1 - \phi + \kappa \phi } $$

where $D_{s}$ and $D_{l}$ are diffusivities in the polymer and liquid within pores, respectively, and $\kappa$ is partitioning between the liquid and solid phase.

### Diffusion within fibers

Diffusion within a fiber consists of two components: axial, in the direction of the fiber axis, and radial, within the fiber cross-section. It was found in^35^ that axial diffusion can be neglected so we omit the corresponding equations here. In Reference^36^, a radial 1D finite element was formulated, where the fiber is represented by a line composed of segments aligned on the fiber axis, with two common points. In order to represent radial diffusion within the fiber and volume belonging to the common point, we introduced radial 1D element as fictitious element in the FE mesh representation. As described in^35^, a radial 1D element consists of two nodes: node 1 is at the symmetry axis of the fiber, while node 2 is at the fiber surface. As an approximation, it is considered that a node of the 3D continuum, closest to node 2 of the fiber, has the same concentration as node 2. A 1D radial element can be divided into subelements which improves accuracy of the numerical solution^36^, since the radial concentration profile is nonlinear. The mass balance equations of the 2-node 1D FE element, used in our computational model, is given in^36^ and also provided in Supplementary material.

### Fundamental equations for CSFE

**Figure 2:**
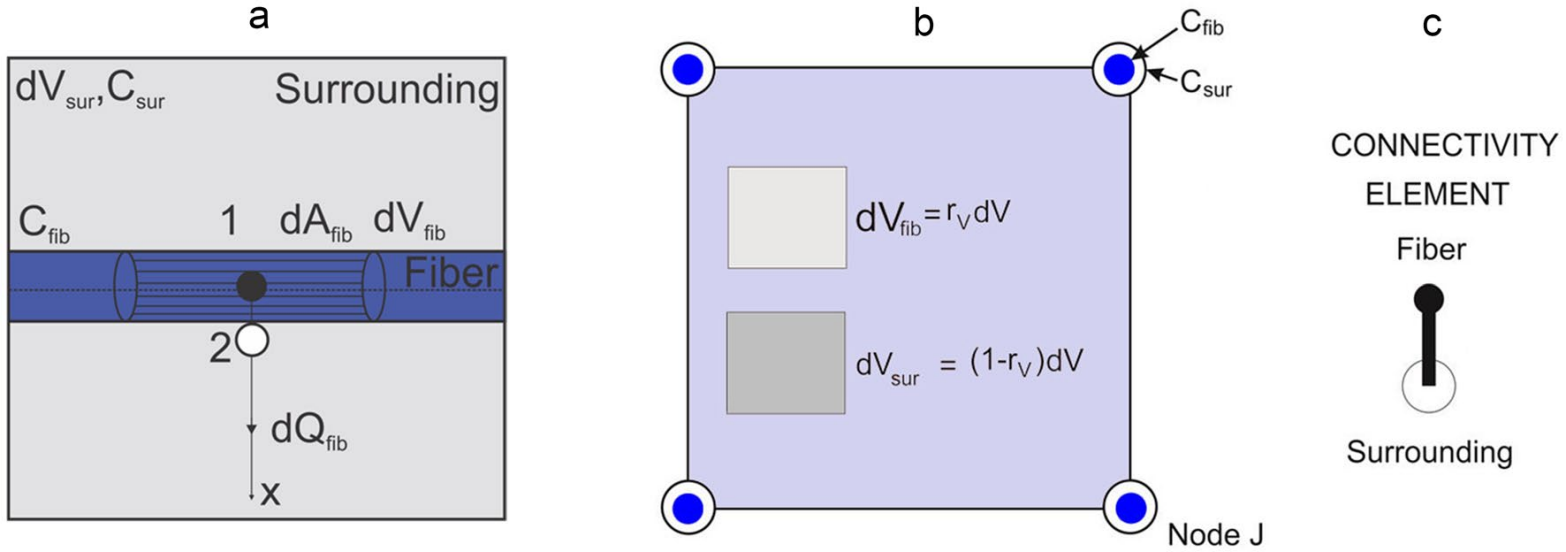
(**a**) Diffusion from fiber surface *dA*_*fib*,_ which corresponds to the fiber volume *dV*_*fib*_ and total volume *dV*; *dV*_*sur*_ is the volume occupied by the surrounding medium, (**b**) representation of composite smeared finite element (CSFE) with two domains: fiber and surrounding^35^, (**c**) 1D connectivity element in CSFE.

Composite smeared finite element (CSFE) for modeling diffusion within a fiber network and the surrounding used here is in analogy with representation of mass transport within a capillary system and surrounding tissue^37–41^. Since axial diffusion within fibers can be neglected, we here only present formulation of the radial diffusion in the smeared concept. Considering diffusion through a fiber^29^, the elementary area of the surface of the fiber wall $dA_{fib}$ can be related to the elementary volume $dV_{fib}$ and further to the elementary total volume $dV$, Fig. 2a. The most fundamental equation we are using in our smeared models, where the discrete fiber surface is smeared over the volume of the continuum, is:

3 $$ dA_{fib} = r_{AV} dV_{fib} = r_{AV} r_{V} dV $$

where $r_{AV}$ is the surface ratio (fiber area-to-volume ratio) and $r_{V}$ is fiber density (the fibers’ volumetric ratio within the surrounding fluid). With assumptions discussed in^35^, diffusive transport between fibers and tissue can be performed by discretizing the continuum only. The parameters of the model, assigned to each continuum node J, are the volumetric ratio of fibers $\left( {r_{V} } \right)_{J}$, the surface ratio $\left( {r_{AV} } \right)_{J}$, mean radius of fibers ($R_{J}$), drug diffusion coefficient $D_{J}$ within fibers and partition coefficient $P_{J}$ at the fiber surfaces.

The CSFE consists of the fiber and the surrounding domain (Fig. 2b), coupled by the connectivity elements at each FE node (Fig. 2c). The balance equations of the 2-node connectivity elements can be written in the form (S.7), with the nodal values of the fiber and surrounding, and the cross-sectional area equal to $\left( {r_{AV} r_{V} V} \right)_{J}$. The volume $V$ of the element is occupied by the fiber domain $r_{V} V$ and by the surrounding medium $(1 - r_{V} )V$. In the case of a straight fiber, the surface ratio is $r_{AV} = 4/D_{fib}$, where $D_{fib}$ is diameter of the fiber. In the model of the PCL/PLGA/PCL scaffold, we use normal distribution function for the fiber radius R, with: R_min_ = 1.0 µm, R_max_ = 4.0 µm, R_mean_ = 2.5 µm to calculate diameters of fibers in each FE node of smeared finite elements.

### Correction functions for CSFE

Composite smeared finite element, despite theoretically correct foundation, doesn’t provide exactly the same overall mass transport to (and from) tissue when compared to a true 3D model. This is because the smeared concept has an averaging feature in each of the representative small domain surrounding the considered FE nodal point. In order to improve accuracy of the CSFE, a field of correction functions for diffusivity through the capillary walls of smeared models, which provides better accuracy of robust smeared models, is introduced in^40^. The same methodology is used here: CSFE for coupled fiber network system is enhanced by using a correction function.

**Figure 3:**
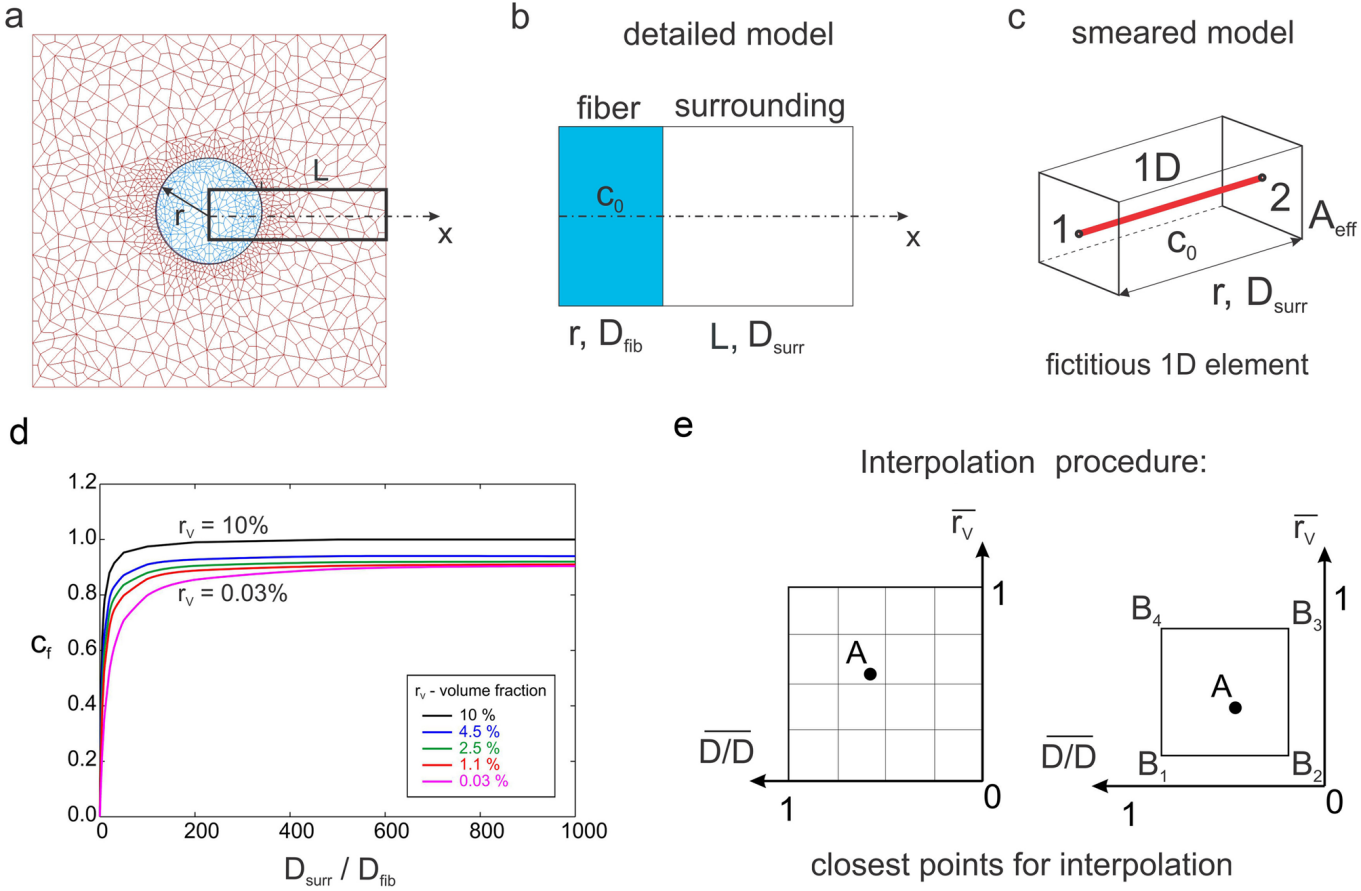
Detailed and smeared 2D model of a 2D diffusion within a fluid surrounding nanofiber, according to^40^ (**a**) Fiber with the radius *r* and concentration C_0_ , and surrounding fluid discretized by continuum Fes. (**b**) Enlarged area of detailed model with fiber and surrounding fluid. (**c**) Smeared model representation of 1D diffusion between points 1 and 2 through the fiber surface area A_eff_. (**d**) Correction function (c_f_) for diffusion coefficient in fiber domain of smeared model—dependence on D_surr_/D_fib_ ratio, for models with different volume fractions (r_V_) of fibers: (**e**) description of interpolation procedure used for calculation of the correction factor.

An example which is tested is shown in Fig. 3a–c. We consider a 2D diffusion within a fluid surrounding nanofiber, where fiber is in the middle of the square domain (100 × 100 µm) and normal to the diffusion plane (Fig. 3a). An initial concentration within fiber is C_0_ and there is no flux at the surrounding boundary. The enlarged segment of FE mesh from Fig. 3a is shown in Fig. 3b and represents a detailed model, while corresponding smeared model representation is given in Fig. 3c. It was shown, but not presented here, that there is difference in overall mass transport comparing smeared and detailed model, since smeared model cannot capture the concentration gradients in the vicinity of the fibers. The difference depends on the model parameters: ratio of diffusion coefficient in fiber (D_fib_) and surrounding tissue (D_surr_); and volume fraction of fibers within surrounding domain $r_{V}^{{}}$, so, diffusion coefficient within the fiber is calculated as:

4 $$ D_{fib}^{smeared} = c_{f} (D_{surr}^{{}} /D_{fib}^{{}} ,r_{V}^{{}} ) \cdot D_{fib}^{true} $$

where *c*_*f*_ is the correction function. The function is determined under the condition that the mass release from the fiber to the surrounding is the same for the detailed and smeared model. The table of the correction function values, in the form displayed in Fig. 3d, expressed by:

5 $$ c_{f}^{ij} = c_{f} ((D_{surr}^{{}} /D_{fib}^{{}} )^{i} ,r_{V}^{j} ) $$

is implemented in our in-house CAD software for pre- and post- processing. Following procedure presented in^40^, we determine the discrete values within the following ranges of parameters: r_v_ = {0.03, 1.1, 2.5, 4.5, 10.0} and D_surr_/D_fib_ = {0.1, 1, 10, 100, 500, 1,000}. Then, for a given values of r_v_ and D_surr_/D_fib_ we calculate the corresponding correction factor. Finally, parameters r_v_ and D_surr_/D_fib_ are normalized by the maximum values producing the range [0,1], and table of correction factors (2D domain in Fig. 3d) is mapped to the unit quad (Fig. 3e). Interpolation for correction factors (values of the correction function) is performed according to procedure presented in^40^, where we find interpolation coefficient for each of points B_1_, B_2_, B_3_, B_4_. The calculated value is then used in 1D connectivity element of the smeared model to determine the corresponding value of the wall diffusion coefficient.

## Numerical and experimental results of drug release

**Figure 4:**
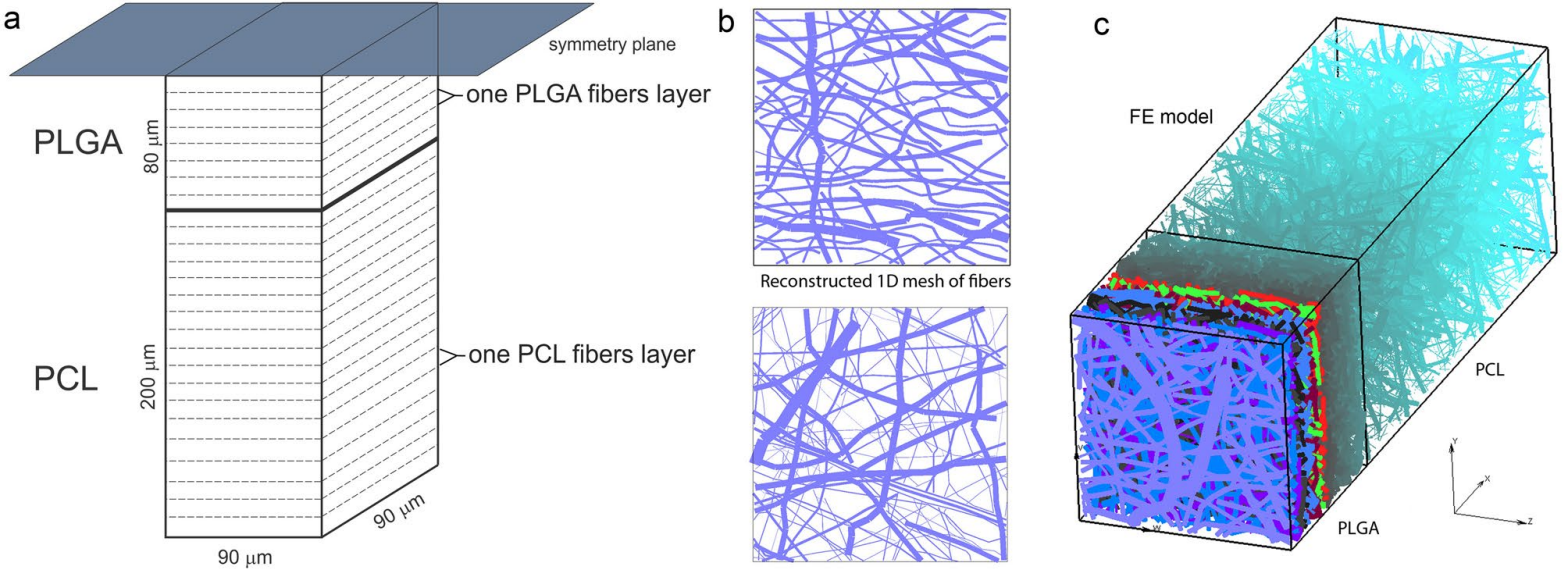
(**a**) Configuration of the finite element (FE) model of PCL/PLGA/PCL scaffold, configuration and geometry of FE model. (**b**) Reconstructed 1D mesh of fibers from SEM imaging of one layer of PLGA and PCL fibers (scan bar 20 μm). (**c**) 3D FE model of PLGA/PCL 3-layer model, with symmetric conditions applied to the model.

A complete PCL/PLGA/PCL scaffold has dimensions of 3 cm × 4 cm, with thickness of 560 µm. The three‐layered fibrous scaffold has layer of PLGA of 160 µm in-between two 200 µm thick PCL layers. The morphology of the nanofiber mats was examined by scanning electron microscopy (SEM), Fig. 4. The network of fibers is reconstructed from an SEM image of 90 µm × 90 µm using indoor software, with the assumption that fibers within PLGA and PCL layers are considered to be ideal cylinders. Reconstructed mesh of fibers serves as input for first computational model we generated: detailed FE model with 1D radial elements. The second PCL/PLGA/PCL model is generated by using CSFE consisted of two different domains: fiber and surrounding domain.

### Preparation and numerical simulation of detailed FE models

Simplified FE model of PCL/PLGA/PCL scaffold is generated according two basic assumptions: symmetry and homogenous distribution. On one hand, since PLGA layer is surrounded by two dimensionally identical PCL layers, we can include symmetry condition and model just one half of the three-layer scaffold (on one side of symmetry plane). One another hand, by assuming homogenous distribution (or repetition) of one small domain of the fibers we can consider just one layer with dimensions of 90 µm × 90 µm. Thus, the size of the simplified FE model is: 280 µm × 90 µm × 90 µm.

Following procedure presented in^35^, we generated two mats of fibers, for both PLGA and PCL layers. Mats of fibers are generated by randomly duplicating and displacing the generated layer of 1D fibers into the longitudinal direction of the modeling domain, (Fig. 4b,c). The 3D FE mesh is composed of 70,785 nodes and 65,536 elements; the number of radial 1D elements is around 14,580 for the considered examples. 3D FE mesh of the surrounding in PLGA layer consists of so-called “immersed” points which take into account effects of position and orientation of the fibers on overall diffusion transport in surrounding fluid medium. The same principle is used for PCL layer according assumption that degradation of PCL is very slow (6 months) and diffusion through PCL fiber is negligible.

The diffusion coefficient of RhB in pore space (space between fibers) is assumed to be as in water, and the diffusion coefficient of RhB within the fibers (fiber with impregnated drug inside) is *D*_fiber_ = 4 × 10^−10^ cm^2^/s, which is taken from^45^. The time period of simulation was 75 days (15 time steps with 5 days each). Concentration of the RhB in the PLGA fiber is uniform and initially equal to C_0_. At the outer boundary of the scaffold, where mass release is measured, we assume infinite reservoir with prescribed C = 0 concentration.

### Application of smeared modeling for drug transport in PLGA/PCL model

**Figure 5:**
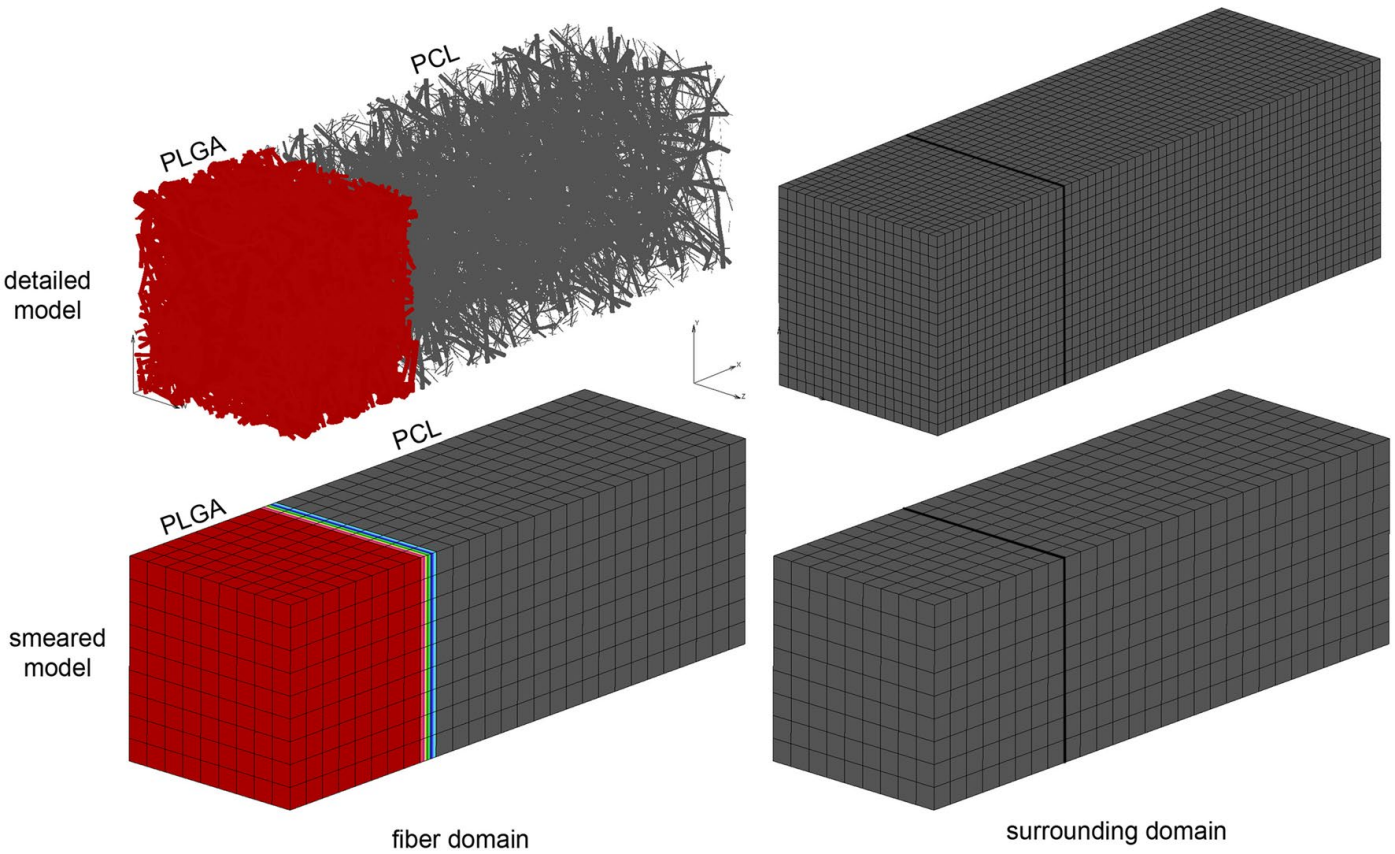
Three-layered PLGA/PCL scaffold modeled using a detailed model with the mesh of fibers (top) and a smeared composite finite element (bottom). The numerical model consists of fiber (left) and surrounding (right) domain.

The smeared model consists of two domains: fiber domain (equivalent domain of fibers) and surrounding domain (equivalent “pore” space surrounding fibers). The input parameters of the model are: volume fraction of fibers in PLGA and PCL layers, diffusion coefficient within PLGA fibers, for 24 wt% 65:35 emulsion, diffusion coefficient of drug within the surrounding domain, coefficient of hydrophobicity (partitioning) at the fiber surface, and mean diameter of PLGA/PCL fibers. We used the detailed and corresponding smeared model for drug transport analysis, which are both shown in Fig. 5. Smeared FE model consists of 2,900 nodes and 2,268 elements, which reduces the number of equations to be solved around 20 times compare to detailed model.

The presence of fibers in the system and attractive forces between fibers and diffusion molecules reduces diffusion coefficient in surrounding medium^46^. The equivalent diffusion coefficient in the surrounding domain is, therefore, calculated using a numerical homogenization procedure according to^46^ and is found to be D_liquid_ = 0.004 µm^2^/s for PLGA and D_liquid_ = 0.006 µm^2^/s for PCL layer. The parameters used in composite smeared finite element (CSFE) model are: volume fraction of fibers is $r_{V}$ = 0.4223, mean diameter of fibers is D = 2.5 µm, diffusion coefficient within PLGA domain: D_wall_ = 0.04 µm^2^/s, equivalent diffusion coefficient in surrounding domain is D_liquid_ = 0.004 µm^2^/s; and partitioning is P = 10^6^.

### Comparison of numerical and experimental results

Assumptions used for smeared model are: fiber domain is active only in PLGA layer. There is no connection between fiber domains in PLGA and PCL layer. Drug leaves fiber domain of PLGA and enters the surrounding of PLGA layer, further goes to the surrounding of PCL and finally leaves the scaffold at the front boundary. We assumed, due to slow degradation of PCL fibers, that there is no diffusion through the fiber domain of PCL layer, and no transport through connectivity elements connecting the smeared fiber and surrounding liquid domain.

Among the parameters affecting the process partitioning is the most important factor influencing the release kinetics. It is known that the hydrophobicity of RhB is higher than hydrophobicity of the Span 80/RhB complex^47^. Also, when using pure RhB molecule, degradation has to be taken into account, according to^14^. Parameters used in the smeared model are as follows: volume fraction of fibers in scaffold is r_V_ = 0.218, mean diameter of fiber is 5 µm with inclusion of fiber distribution function, and diffusion coefficient of RhB is the same for porous fiber and surrounding fluid D_wall_ = D_liquid_ = 0.04 µm^2^/s. Parameters for degradation are the same as in detailed model: partitioning coefficient is P = 10^6^, degradation rate constants: K = Kw = 2.5e−007, α = 1.714, initial porosity = 0, D_plga_ = 0 µm^2^/s, D_liquid_ = 0.04 µm^2^/s. The computational model matches the experimental release curves for partitioning coefficients of P = 10^6^ for RhB.

**Figure 6:**
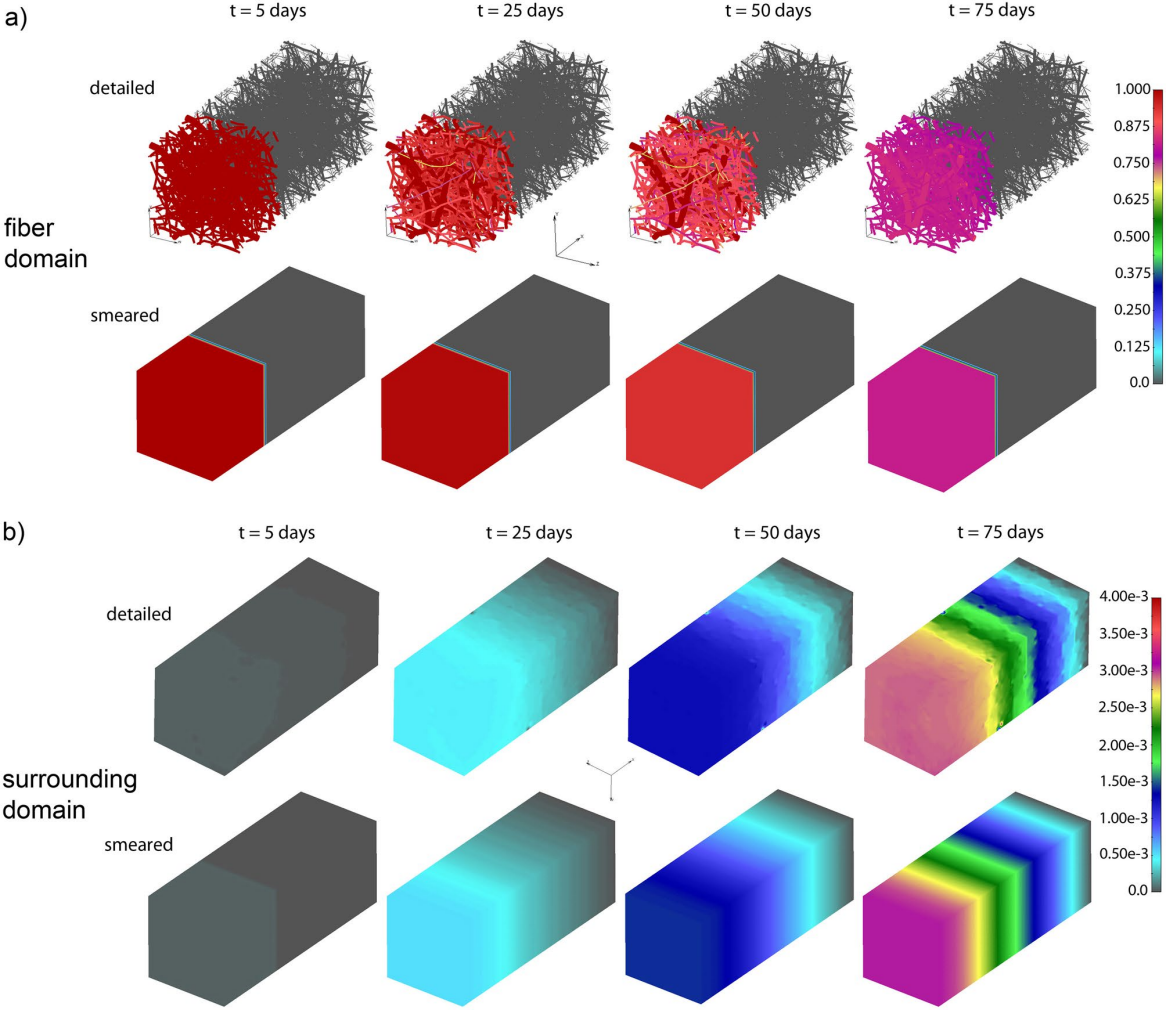
Three-layered PLGA/PCL scaffold concentration field for fiber and surrounding domain of the detailed and smeared model, for the diffusion of RhB within the PLGA layer.

Concentration fields for both detailed and smeared models of PCL/PLGA/PCL scaffold are shown in Fig. 6, for within the fibers and the surroundings, and within a period of 75 days. A very small difference in results between those two models proves the accuracy of smeared model and its applicability for the prediction of drug transport from layer-by-layer scaffolds.

**Figure 7:**
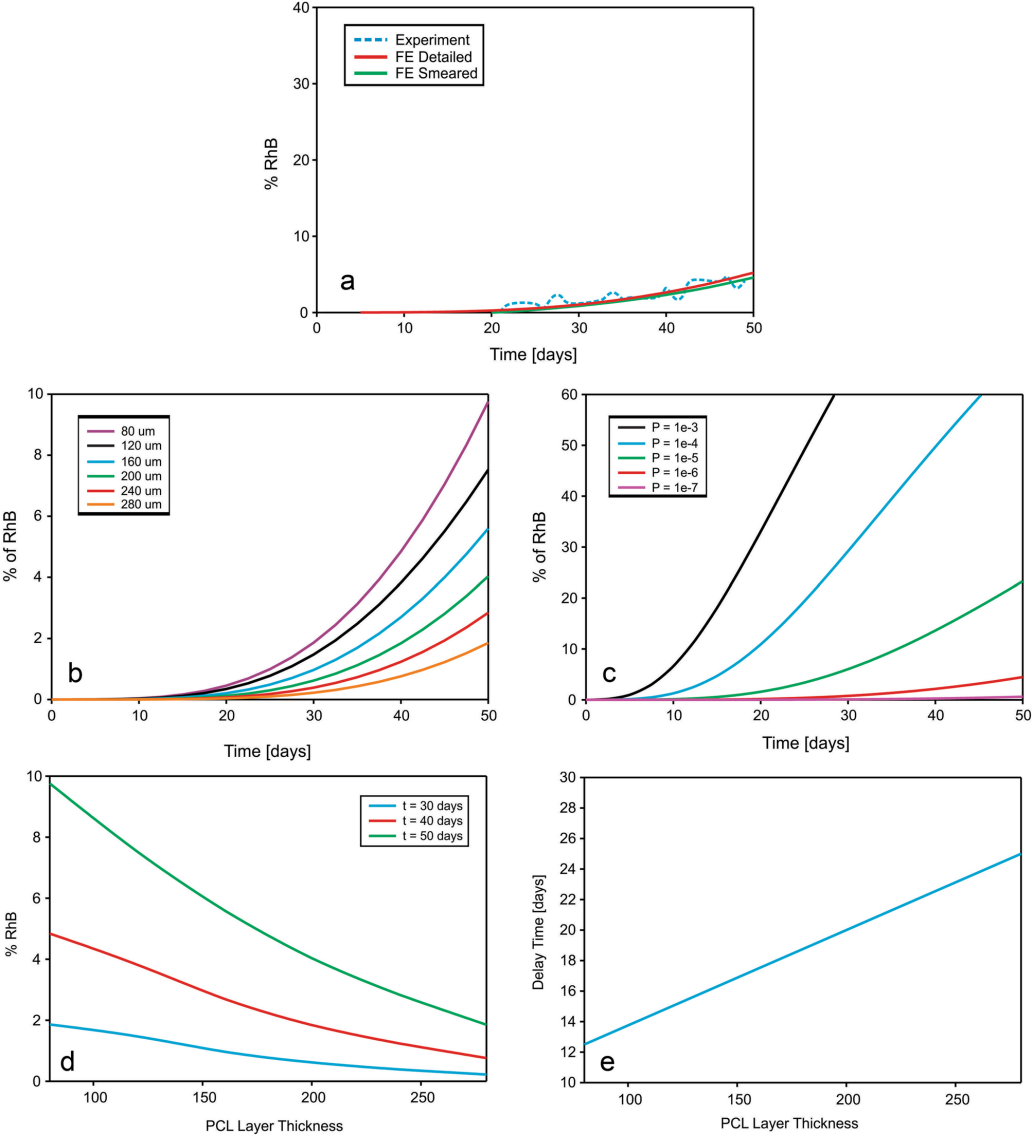
(**a**) Cumulative release vs. time for RhB complex impregnated and for 24 wt% 65:35 PLGA. Experimental curve (dashed) and results of FE simulation obtained using the true (detailed) and smeared model of PLGA/PCL fibers. Parameter-based prediction of the computational model: (**b**) release dependence on layer thickness. (**c**) Effect of drug partitioning (hydrophobicity). (**d**) Released drugs vs. thickness of PCL layer mats for various times. (**e**) Delay of initial burst due to thickness of PCL layer.

Diagrams of cumulative mass release obtained from the experiment and FE simulations of PLGA/PCL (both detailed and smeared models) are given in Fig. 7a. It can be seen that smeared model is accurate as detailed model and can be used for mass release prediction in PCL/PLGA/PCL three‐layer scaffold.

Additionally, numerical model can be used for optimization of the scaffold and prediction of the mass release process for different input parameters. Instead of producing new experimental configuration of the scaffold and measuring release process (usually takes months or two), the numerical FE model is capable of predicting the amount of drug and speed of the process for different characteristics of the scaffold for less than a few minutes. Cumulative mass release vs. time diagram for RhB complex and for different thicknesses of the PCL layer is shown in Fig. 7b. RhB is encapsulated within Span 80 layer. In the absence of Span 80, there would be no hydrophobic layer, and RhB will come out from the PLGA fiber immediately, which was detected by experiment and confirmed by this numerical simulation. Computational model can also predict release profiles for different values of partitioning coefficient. RhB release for different partitioning P is shown in Fig. 7c. The same results are obtained using either smeared or detailed numerical model.

As can be seen from Fig. 7d, percentage of RhB released from the fibers is inversely related to the thickness of PCL layer: increase of the PCL layer thickness reduces the amount of released molecule. Delay of initial burst, shown in Fig. 7e, are displayed under the assumption that the first occurrence of the drug at outlet of FE model happens when mass at outlet reach 0.1 wt% of the initial mass of RhB in PLGA layer.

## Discussion

The use of water-in-oil (w/o) emulsion electrospinning to incorporate hydrophilic drugs into co-electrospun fibers was reported by^48^, with aim to achieve prolonged and to avoid burst drug release. In this work, w/o emulsion electrospun fibers (with average diameter 2.50 ± 1.66 µm) were successfully fabricated using Span 80 nonionic surfactant as an emulsifier, which facilitated electrospinning of the aqueous phase with encapsulated hydrophilic RhB drug (lower viscosity), and oily phase with larger viscosity (chloroform/DMF solution of hydrophobic PLGA copolymer).

Our numerical model gives the same prediction as detected in references^14,47^. In^47^ “Rhodamine B molecule was uniformly encapsulated in the core–shell electrospun fiber mat, and could not be released until PLGA started to degrade”. As shown in Fig. 4 of reference^47^, the first occurrence of Rhodamine B molecule is detected approximately 200 h after the mat is immersed in PBS. Similarly, as shown in Fig. 5 of reference^14^, notable degradation of the RhB/PLGA electrospun nanofiber mat is detected after 50 days, which is consequence of exposure of mat to the PBS solution which allows partial degradation of PLGA.

In^35^ we analyzed degradation and hydrophobicity effects of (PLGA/RhB/Span 80). In this work we used the RhB molecule which has much larger hydrophobicity leading to slower degradation of the (PLGA fiber/RhB). In this fibrous scaffold, initial release from the scaffold is detected after 500 h, which is because of the presence of the additional hydrophobic PCL layer. PCL is usually more hydrophobic than PLGA and hence degrades at a much slower rate than copolymer. The release tests also showed that the thickness of the fibrous PCL layer critically affects the amount of the initial burst of RhB and number of stacked layers, as reported in^27^.

Additionally, due to slow degradation and large density of PCL fiber layers, the first occurrence of drugs out of the scaffold is detected after 21 days, which is the time required for wound healing after surgery. The final prediction of the computational model is that 9% of initially embedded drug will be released after 60 days, which is also detected by experiment. Following this findings, we may expect that computational model can predict mass release even for longer time periods.

As was shown in Fig. 7b, our computational model is able to give accurate mass release prediction for different thicknesses of PCL layer. Results presented in Fig. 7d is in accordance with experimental results with layer-by-layer PEO/PCL mats published in^27^, where the release of the drug is linearly dependent with respect to the thickness of the porous electrospun PCL mat. The same approach can be used in order to predict releases from models which differs in average diameters of the fibers in the system, volume fraction of fibers in the system, degradation time and partitioning (hydrophobicity) of the drug used. Numerical model, therefore, can be used as prediction tool, and can simplify process to choose an appropriate polymer for the corresponding type of postoperative treatment and therapy, and can reduce health issues which can occur by experimenting with real and toxic cancer treatment drugs.

Previous research with multi-layer scaffolds has developed controlled release materials and methods where drug release begins immediately after the first day^49–51^. Our unique scaffold system can be used for localized drug release and potential post-surgical cancer treatment twenty-one days after surgery. The most significant precedence of our method is that the release profiles can follow surgical requirements for wound healing and subsequently deposed of therapeutics drugs in the desired time frame at precise positions. To our knowledge, such a sampling technique has not yet been employed in a localized drug delivery in solid cancer recurrence and perhaps find a new clinical application.

## Conclusions

The combination of emulsion and sequential electrospinning generated three‐layered fibrous scaffold is capable of providing a prolonged release of different hydrophilic drugs such as RhB. Scaffold consists of three layers, where the PCL layer is sandwiched in between two PLGA layers.

Recently introduced FE computational models (detailed with the radial elements for drug release from fibers, and smeared model) are used to simulate drug transport from drug loaded PCL/PLGA/PCL layer-by the layer scaffold. Both smeared and detailed models can be used to match results obtained by experiments. Additionally, both models can predict the delay time and the release rate in terms of thickness of the PCL layer. Presented smeared and detailed methodology can be used for mass release prediction in different implants and scaffolds, with different material characteristics, such as: porosity, drug diffusion coefficient, hydrophobicity and degradation rate.

Accurate prediction of drug release can be achieved by applying both detailed and smeared models. However, the smeared computational model (CSFEs) is as accurate as detailed model, but more efficient and simpler to be used in practical applications^35^. This model is particularly attractive for drug transport within layer-by-layer design of fibrous scaffolds used in medical purposes.

## Supplementary information

Supplementary Information

## Data Availability

No datasets were generated or analysed during the current study.

## References

[1] Kenawy ER (2002). Release of tetracycline hydrochloride from electrospun poly (ethylene-co-vinylacetate), poly (lactic acid), and a blend. J. Control. Release.

[2] Luo X (2012). Antitumor activities of emulsion electrospun fibers with core loading of hydroxycamptothecin via intratumoral implantation. Int. J. Pharm..

[3] Katti DS, Robinson KW, Ko FK, Laurencin CT (2004). Bioresorbable nanofiber-based systems for wound healing and drug delivery: Optimization of fabrication parameters. J. Biomed. Mater. Res. B Appl. Biomater..

[4] Li J (2018). Locally deployable nanofiber patch for sequential drug delivery in treatment of primary and advanced orthotopic hepatomas. ACS Nano.

[5] Li J (2017). Highly bioadhesive polymer membrane continuously releases cytostatic and anti-inflammatory drugs for peritoneal adhesion prevention. ACS Biomater. Sci. Eng..

[6] Raizada A, Bandari A, Kumar B (2010). Polymers in drug delivery: A review. Int. J. Pharm. Res. Dev..

[7] Dash TK, Konkimalla VB (2012). Poly-є-caprolactone based formulations for drug delivery and tissue engineering: A review. J. Control Release..

[8] Gillies ER, Frechet JM (2005). Dendrimers and dendritic polymers in drug delivery. Drug Discov. Today.

[9] Rai A, Senapati S, Saraf SK, Maiti P (2016). Biodegradable poly(e-caprolactone) as a controlled drug delivery vehicle of vancomycin for the treatment of MRSA infection. J. Mater. Chem. B.

[10] Singh NK (2013). CNT induced β-phase in polylactide: Unique crystallization, biodegradation, and biocompatibility. J. Phys. Chem. C.

[11] Maiti P, Yadav PJP (2008). Biodegradable nanocomposites of poly(hydroxybutyrate-co-hydroxyvalerate): The effect of nanoparticles. J. Nanosci. Nanotechnol..

[12] Maiti P, Batt CA, Giannelis EP (2007). New biodegradable polyhydroxybutyrate/layered silicate nanocomposites. Biomacromol.

[13] Gentile P, Chiono V, Carmagnola I, Hatton PV (2014). An overview of poly(lactic-co-glycolic) acid (PLGA)-based biomaterials for bone tissue engineering. Int J. Mol. Sci..

[14] Zhu X, Braatz RD (2015). A mechanistic model for drug release in PLGA biodegradable stent coatings coupled with polymer degradation and erosion. J. Biomed. Mater. Res. A.

[15] Mano JF, Sousa RA, Boesel LF, Neves NM, Reis RL (2004). Bioinert, biodegradable and injectable polymeric matrix composites for hard tissue replacement: State of the art and recent developments. Compos. Sci. Technol..

[16] Lin HR, Kuo CJ, Yang CY, Shaw SY, Wu YJ (2002). Preparation of macroporous biodegradable PLGA scaffolds for cell attachment with the use of mixed salts as porogen additives. J. Biomed. Mater. Res. A.

[17] Sackett CK, Narasimhan B (2011). Mathematical modeling of polymer erosion: Consequences for drug delivery. Int. J. Pharm..

[18] Makadia HK, Siegel SJ (2011). Poly lactic-co-glycolic acid (PLGA) as biodegradable controlled drug delivery carrier. Polymers (Basel)..

[19] Siepmann J, Geopferich A (2001). Mathematical modeling of bioerodible, polymeric drug delivery systems. Adv. Drug Deliv. Rev..

[20] Singh NK (2012). Nanostructure controlled anti-cancer drug delivery using poly(ε-caprolactone) based nanohybrids. J. Mater. Chem..

[21] Kweon HY (2003). A novel degradable polycaprolactone networks for tissue engineering. Biomaterials.

[22] Bose S, Roy M, Bandyopadhyay A (2012). Recent advances in bone tissue engineering scaffolds. Trends Biotechnol..

[23] Woodruff MA, Hutmacher DW (2010). The return of a forgotten polymer–polycaprolactone in the 21st century. Prog. Polym. Sci..

[24] Zeng J (2003). Biodegradable electrospun fibers for drug delivery. J. Control Release.

[25] Zeng J (2005). Influence of the drug compatibility with polymer solution on the release kinetics of electrospun fiber formulation. J. Control Release.

[26] Gao H, Gu Y, Ping Q (2007). The implantable 5-fluorouracil-loaded poly(l-lactic acid) fibers prepared by wet-spinning from suspension. J. Control Release.

[27] Kim GH, Yoon H, Park YK (2010). Drug release from various thicknesses of layered mats consisting of electrospun polycaprolactone and polyethylene oxide micro/nanofibers. Appl. Phys. A.

[28] Yoon H, Kim GH (2012). Layer-by-layered electrospun micro/nanofibrous mats for drug delivery system. Macromol. Res..

[29] Petlin DG (2017). A fiber distribution model for predicting drug release rates. J. Control Release.

[30] Jiang YN, Mo HY, Yu DG (2012). Electrospun drug-loaded core–sheath PVP/zein nanofibers for biphasic drug release. Int. J. Pharm..

[31] Li L (2011). Electrospun poly (ɛ-caprolactone)/silk fibroin core-sheath nanofibers and their potential applications in tissue engineering and drug release. Int. J. Biol. Macromol..

[32] Illangakoon UE (2014). Fast dissolving paracetamol/caffeine nanofibers prepared by electrospinning. Int. J. Pharm..

[33] Grkovic M (2017). Improvement of mechanical properties and antibacterial activity of crosslinked electrospun chitosan/poly (ethylene oxide) nanofibers. Compos. B Eng..

[34] Radisavljevic A (2018). Cefazolin-loaded polycaprolactone fibers produced via different electrospinning methods: Characterization, drug release and antibacterial effect. Eur. J. Pharm. Sci..

[35] Milosevic M (2018). Computational model for drug release from PLGA implant. Materials (Basel, Switzerland).

[36] Kojic M, Milosevic M, Simic V, Stojanovic D, Uskokovic P (2017). A radial 1D finite element for drug release from drug loaded nanofibers. J. Serb. Soc. Comput. Mech..

[37] Kojic M (2017). A composite smeared finite element for mass transport in capillary systems and biological tissue. Comput. Methods Appl. Mech. Eng..

[38] Kojic M (2018). Multiscale smeared finite element model for mass transport in biological tissue: From blood vessels to cells and cellular organelles. Comput. Biol. Med..

[39] Kojic M (2018). Smeared concept as a general methodology in finite element modeling of physical fields and mechanical problems in composite media. J. Serb. Soc. Comput. Mech..

[40] Milosevic M (2018). Correction function for accuracy improvement of the composite smeared finite element for diffusive transport in biological tissue systems. Comput. Methods Appl. Mech. Eng..

[41] Kojic M (2019). Smeared multiscale finite element model for electrophysiology and ionic transport in biological tissue. Comput. Biol. Med..

[42] Kojic M (2018). PAK-FE program for structural analysis, fluid mechanics, coupled problems and biomechanics.

[43] Ru CH (2015). Suspended, shrinkage-free, electrospun PLGA nanofibrous scaffold for skin tissue engineering. ACS Appl. Mater. Interfaces.

[44] Kojic M, Filipovic N, Stojanovic B, Kojic N (2008). Computer Modelling in Bioengineering—Theory, Examples and Software, NJ, USA.

[45] Ruiz-Esparza GU (2014). Polymer nanoparticles enhanced in a cyclodextrin complex shell for potential site- and sequence-specific drug release. Adv. Funct. Mater..

[46] Ziemys A (2001). Hierarchical modeling of diffusive transport through nanochannels by coupling molecular dynamics with finite element method. J. Comput. Phys..

[47] Liao Y, Zhang L, Gao Y, Zhu ZT, Fong H (2008). Preparation, characterization, and encapsulation/release studies of a composite nanofiber mat electrospun from an emulsion containing poly (lactic-co-glycolic acid). Polymer.

[48] Mirabedini A (2018). Developing Novel Spinning Methods to Fabricate Continuous Multifunctional Fibres for Bioapplications.

[49] Han D (2019). Multi-layered core-sheath fiber membranes for controlled drug release in the local treatment of brain tumor. Sci. Rep..

[50] Ramachandran R (2017). Theranostic 3-Dimensional nano brain-implant for prolonged and localized treatment of recurrent glioma. Sci. Rep..

[51] Nagiah N (2020). Development of tripolymeric triaxial electrospun fibrous matrices for dual drug delivery applications. Sci. Rep..

