# Author Correction: Preparation and modeling of three‐layered PCL/PLGA/PCL fibrous scaffolds for prolonged drug release

**DOI:** 10.1038/s41598-021-95377-w

**Published:** 2021-08-02

**Authors:** Miljan Milosevic, Dusica B. Stojanovic, Vladimir Simic, Mirjana Grkovic, Milos Bjelovic, Petar S. Uskokovic, Milos Kojic

**Affiliations:** 1 Bioengineering Research and Development Center BioIRC Kragujevac, Prvoslava Stojanovica 6, Kragujevac, 34000 Serbia; 2 Belgrade Metropolitan University, Tadeusa Koscuska 63, Belgrade, 11000 Serbia; 3 Faculty of Technology and Metallurgy, University of Belgrade, Karnegijeva 4, Belgrade, 11000 Serbia; 4 Department for Minimally Invasive Upper Digestive Surgery, Clinical Center of Serbia, Hospital for Digestive Surgery - First Surgical Hospital, Dr Koste Todorovica 66, Belgrade, 11000 Serbia; 5 The Department of Nanomedicine, Houston Methodist Research Institute, 6670 Bertner Ave., R7 117, Houston, TX 77030 USA; 6 Serbian Academy of Sciences and Arts, Knez Mihailova 35, Belgrade, 11000 Serbia

Correction to: *Scientific Reports* 10.1038/s41598-020-68117-9, published online 07 July 2020

The original version of this Article contained an error in the Acknowledgments section.

“This paper is supported by the SILICOFCM project that has received funding from the European Union’s Horizon 2020 research and innovation programme under grant agreement No 777204. This research was also funded by Ministry of Education and Science of Serbia, grants OI 174028, III 41007, III 45019 and PROMIS, Science Fund of the Republic of Serbia. The authors acknowledge support from the City of Kragujevac, Serbia.”

now reads:

“This paper is supported by the SILICOFCM project that has received funding from the European Union’s Horizon 2020 research and innovation programme under grant agreement No 777204. This research was also funded by Ministry of Education and Science of Serbia, grants OI 174028, III 41007, III 45019. The authors acknowledge support from the City of Kragujevac, Serbia.”

The original Article has been corrected.

