# Supplementary material for: Preparation and modeling of three‐layered PCL/PLGA/PCL fibrous scaffolds for prolonged drug release
Source: Sci Rep. 2020 Jul 7;10:11126. doi: 10.1038/s41598-020-68117-9 (PMC7341868; doi:10.1038/s41598-020-68117-9)
Supplement: Supplementary file 1 — Supplementary Information [file 41598_2020_68117_MOESM1_ESM.docx]

**Preparation and modeling of three‐layered PCL/PLGA/PCL ﬁbrous scaffolds for prolonged drug release**

Miljan Milosevic, Dusica B. Stojanovic, Vladimir Simic, Mirjana Grkovic, Milos Bjelovic, Petar S. Uskokovic and Milos Kojic

**Supplementary material**

Materials and Methods

The vertical electrospinning device (Linari Engineering, Italy) used for implant preparation consisted of a syringe pump (R-100 E, RAZEL Scientiﬁc Instruments), a high-voltage DC power supply generator (PCM50P120, Spellman USA, (Fig. S1).


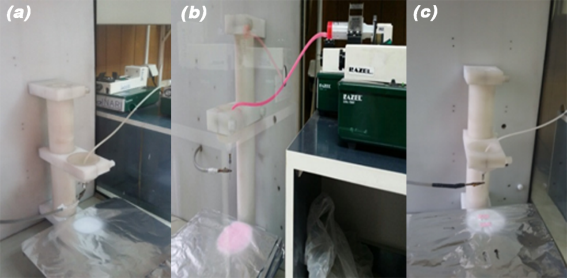


**Fig. S1** The vertical electrospinning device used for implant preparation of (a) PCL layer, (b) PLGA layer and (c) (PCL/PLGA) layers

The morphology of each layer is presented in Figs. S2 and S3 and cross-sectional SEM images of tri-layered fibrous implant in Fig. S3c.


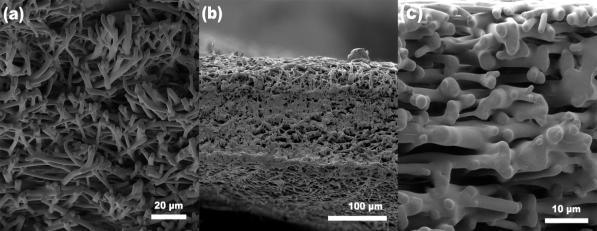


**Fig. S2** SEM images showing morphologies of the nanofiber mats of the PLGA layer (a-c)


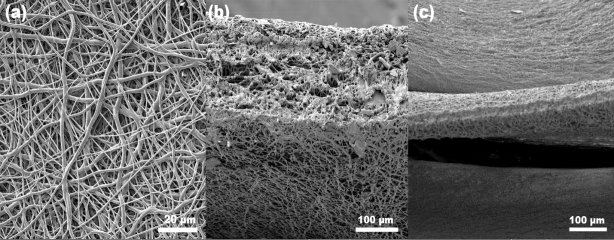


**Fig. S3** SEM images showing morphologies of the nanofiber mats of the PCL layer (a, b) and tri-layered fibrous implant (c)

Computational Models

In this section, we summarize the basic equations from [35,36] for diffusion and degradation, and formulation of the 1D and composite smeared finite element used to model diffusion within fibers.

A1. Fundamental Equations

The balance equation for diffusion in a 3D space, which is based on Fick’s law, can be written as [44]:

| $-\frac{\partial c}{\partial t}+\frac{\partial}{\partial x}_{i}\left(D_{\text{ij}}\frac{\partial c}{\partial x_{j}}\right)+q=0,\text{ sum on }i,j;\text{ }i=1,2,3$ | (S.1) |
| --- | --- |

where $c$ is concentration, $D_{\text{ij}}$ are diffusion tensor coefficients, and $q$ is a source term. In the case of 1D diffusion, this equation reduces to:

| $-\frac{\partial c}{\partial t}+\frac{\partial}{\partial x}\left(D\frac{\partial c}{\partial x}\right)+q=0$ | (S.2) |
| --- | --- |

where *D* is diffusion coefficient for diffusion along the x-direction. This equation is used as the basic equation for diffusion within nanofibers. The functions which take degradation and hydrophobicity effects into account are taken from [14] and are as follows. The function *D = D(M_w_, φ)*, which takes degradation effect into account can be expressed as:

|  | (S.3) |
| --- | --- |

where $D_{s}$ and $D_{l}$ are diffusivities of the polymer phase and liquid filled pores, respectively, and $\kappa$ is partitioning (measure of hydrophobicity) between the liquid-filled pores and solid PLGA phase. Diffusivity is given by the expression:

| $D_{s}=D_{s0}\left(\frac{M_{w}}{M_{w,0}}\right)^{-\alpha}$ | (S.4) |
| --- | --- |

where *D_s0_* is diffusivity for the initial molecular weight *M_w,0_* and α = 1.714 - experimentally determined coefficient. The molecular weight *M*_w_ and porosity *ϕ* are functions of time *t*, described as:

| $M_{w}=M_{w,0} e^{-k_{w}t}$ | (S.5) |
| --- | --- |

and:

|  | (S.6) |
| --- | --- |

where *k_w_* and *k* are degradation rate constants, taken as 2.5 × 10^−7^ s^−1^, and φ_0_( = 0) is the initial porosity.

**Diffusion within Fibers**

Two components of diffusion within a fiber can be distinguished: Axial, in the direction of the fiber axis, and radial, within the fiber cross-section. This diffusion process of axial diffusion is described by Equation (S.2) in differential form, which can be transformed into the finite element form by a standard Galerkin weighting procedure [44]. The FE balance equations can be written for a time step of size $\Delta t$ and equilibrium iteration *i* as:

| $\left(\frac{1}{\Delta t}\mathbf{M}+\mathbf{K}^{\left(i-1\right)}\right)\Delta\mathbf{C}^{\left(i\right)}=\frac{1}{\Delta t}\mathbf{M}\left(\mathbf{C}^{\left(i-1\right)}-\mathbf{C}^{t}\right)-\mathbf{K}^{\left(i-1\right)}\mathbf{C}^{\left(i-1\right)}$ | (S.7) |
| --- | --- |

where matrices $M_{IJ}$ and $K_{IJ}$ are:

| $M_{IJ}=\int_{L} N_{I}N_{J}AdL,\text{ }K_{IJ}=\int_{L} DN_{I,x}N_{J,x}AdL$ | (S.8) |
| --- | --- |

where *N_I_*, *N_J_* are the interpolation functions, *A* is the fiber cross-sectional area, and *L* is element length; **C** and **C^t^** are nodal concentrations at the end and start of time step, respectively. Note that the balance equation of the form (S.7) can be written for the continuum, using Equation (S.1), with matrices:

| $M_{IJ}=\int_{V} N_{I}N_{J}dV,\text{ }K_{IJ}=\int_{V} D_{ij}N_{I,i}N_{J,j}dV$ | (S.9) |
| --- | --- |

where $V$is the FE volume.

A fiber is represented by a line composed of segments aligned on the fiber axis, with common points. The mass balance equations of the 2-node 1D FE element [36] are of the form (S.7) where matrices $M_{IJ}$ and $K_{IJ}$ are:

|  | (S.10) |
| --- | --- |

If we use more than one radial element, the matrices for the radial subelements can be derived in analytical form as:

| $M_{11}=2\pi\bar{L}\int_{0}^{L} \left(R_{i}+x\right)N_{1}N_{1}dx=2\pi\bar{L}L\left(\frac{1}{3}R_{i}+\frac{1}{12}L\right)$ $M_{12}=2\pi\bar{L}\int_{0}^{L} \left(R_{i}+x\right)N_{1}N_{2}dx=2\pi\bar{L}L\left(\frac{1}{6}R_{i}+\frac{1}{12}L\right)$ $M_{22}=2\pi\bar{L}\int_{0}^{L} \left(R_{i}+x\right)N_{2}N_{2}dx=2\pi\bar{L}L\left(\frac{1}{3}R_{i}+\frac{1}{4}L\right)$ | (S.11) |
| --- | --- |
| $K_{11}=K_{22}=-K_{12}=-K_{21}=\frac{2\pi\bar{L}}{L^{2}}D_{fiber}\int_{0}^{L} \left(R_{i}+x\right)dx=\frac{2\pi\bar{L}}{L}D_{fiber}\left(R_{i}+\frac{L}{2}\right)$ | (S.12) |

where *R_i_* is the radius of node 1 of the current subelement (closer to the axis of symmetry) and *L* is the element length.

**Fundamental Equations for CSFE**

Mass balance FE equations for the fluid (surrounding - domain in which the fibers are immersed) of the CSFE element have the form (S.7) with the element matrices

| $M_{IJ}=\int_{V} \left(1-r_{V}\right)N_{I}N_{J}dV,\text{ }K_{IJ}=\int_{V} \left(1-r_{V}\right)D_{ij}N_{I,i}N_{J,j}dV$ | (S.13) |
| --- | --- |

where r_V_ is volumetric fraction of the fibers and *D_ij_* is the diffusion tensor of the surrounding, respectively. Regarding the connectivity elements, the fundamental is the expression for the flux from the fiber to the surrounding. It can be expressed as:

| $dQ_{fib}=\left[D\left(C_{fib}-PC_{sur}\right)-\frac{R}{6\Delta t}\left(C-C^{t}\right)_{fib}-\frac{R}{3\Delta t}\left(PC-C^{t}\right)_{sur}\right]r_{AV}r_{V}dV$ | (S.14) |
| --- | --- |

where $C_{fib}$, $C_{fib}^{t}$, $C_{sur}$, and $C_{sur}^{t}$ are the fiber and surrounding concentrations at the end and start of time step, respectively; P is partitioning at the fiber-fluid interface, and $R$ is radius of the fiber. The nodal fluxes of a continuum finite element are:

| $Q_{fibI}=\int_{V} N_{I}dQ_{fib}=\int_{V} N_{I}\left(...\right)\left(1-r_{V}\right)dV$ | (S.15) |
| --- | --- |

where terms within the parenthesis (…) follow from Equation (S.14), and $N_{I}$ is the continuum interpolation functions of the element with the volume V. The balance equation for the connectivity element at a continuum node *J* can be expressed using Equation (S.10), where $C_{1}=C_{fib}$, $C_{2}=C_{sur}$ at the node *J* (Fig. 5), and the matrices are:

| $M_{11}=\frac{1}{3}A_{fibI}R_{J},\text{ }M_{12}\text{=}M_{21}=\frac{1}{6}P_{J}A_{fibI}R_{J},\text{ }M_{22}=P_{J}M_{11}\text{ }$ $K_{11}=A_{fib}D_{fibJ},\text{ }K_{22}=-K_{12}=-K_{21}=P_{J} K_{11}$ | (S.16) |
| --- | --- |

where (at node *J*) $P_{J}$ is partitioning coefficient as in Equation (S.14); $D_{fibJ}$ is the fiber diffusion coefficient; $R_{J}$is the fiber radius; and$A_{capJ}$is the fiber surface area belonging to the node *J*, which is:

| $A_{fibJ}=\left(r_{AV}r_{V}\right)_{J}V_{J}$ | (S.17) |
| --- | --- |

with $\left(r_{V}\right)_{J}$,$\left(r_{AV}\right)_{J}$being the volumetric ratio and the area coefficient; and $V_{J}$ is the volume of the continuum, which belongs to the node. The volume $V_{J}$ can be numerically evaluated as:

| $V_{J}=\sum_{elements} \int_{V} N_{J}dV$ | (S.18) |
| --- | --- |

where summation includes all elements containing node *J*. It is important to note that nodes representing one connectivity element have the same spatial position.
